# Soil-applied selenite increases selenium and reduces cadmium in roots of *Moringa oleifera*

**DOI:** 10.1038/s41598-020-77350-1

**Published:** 2020-11-23

**Authors:** Zhiqiu Fu, Gang Liu, Lijuan Du, Luxiang Wang, Hongmei Yan, Benlin Yin, Quanhong Ou

**Affiliations:** 1 School of Physics and Electronic Information, Yunnan Normal University, Kunming, 650500 China; 2 Institute of Quality Standards and Testing Technology, Yunnan Academy of Agricultural Sciences, Kunming, 650223 China

**Keywords:** Biological techniques, Plant sciences, Environmental sciences, Optics and photonics

## Abstract

Deficiency of selenium (Se) will lead to malnutrition and decreased immune function of the body. There is a common phenomenon of Se deficiency in foods. In this study, different concentrations of sodium selenite (Na_2_SeO_3_) were applied to *Moringa oleifera* grownin soil. The purpose was to explore the feasibility of Se biofortification of *M. oleifera* root. The effect of exogenous Se on the accumulation of Se and cadmium (Cd) in the roots of *M. oleifera* was studied by inductively coupled plasma mass spectrometry, and the mechanism of exogenous Se on the accumulation of Se and Cd in the roots was studied by Fourier transform infrared spectroscopy (FTIR) combined with principal component analysis and partial least squares regression analysis. The results showed that Na_2_SeO_3_ significantly affected the accumulation of Se and Cd in the roots (*p* < 0.05). The increase in Se was highest when Na_2_SeO_3_ was around 4.0 mg/kg, which increased by 315% compared with the control. The decrease in Cd was the lowest when Na_2_SeO_3_ was around 2.0 mg/kg, which decreased by 80% compared with the control. The results of FTIR analysis showed that Na_2_SeO_3_ treatment changed the carboxylate, phosphate radical, hemicellulose and protein in roots of *M. oleifera*, while the increase of Se was related to hemicellulose, protein, polysaccharide and lignin, and the decrease of Cd was related to hemicellulose and protein. The results showed that exogenous Se increased the accumulation of Se and inhibited the absorption of Cd. Therefore, the roots of *M. oleifera* can be used in Se biofortified products.

## Introduction

Cd is a toxic element, and the long-term exposure of human body to Cd will lead to many diseases, such as renal insufficiency, osteoporosis and even cancer^1^. For the average non-smoking population, about 90% of Cd exposure generally comes from food consumption^2^. The movement of Cd from soil to plant system is strong, so it is easy to be absorbed by crops and enter the food chain, which can affect the yield and quality of crops. Therefore, reducing the absorption of Cd by crops is the key to control human exposure to Cd. The accumulation of Cd in plants will interfere with normal cell function and metabolism, resulting in a series of adverse reactions, such as growth retardation, inhibition of photosynthesis, inhibition of the synthesis of some enzymes, formation of free radicals and changes in ultrastructure at all levels^3–5^. A large amount of active oxygen generated by Cd through enzymatic and non-enzymatic reactions can cause non-specific oxidation of proteins and membrane lipids or DNA damage^6,7^.

Se is an essential trace element for human body. It plays an important role in the biological processes such as antioxidant response, thyroid hormone production, immune response, which can improve cardiovascular disease, muscle disorder and immunity decline^8^. Se is also involved in the synthesis of glutathione peroxidase and thioredoxin reductase^9^. The human body absorbs Se mainly through food. It is an effective way to supplement Se is by eating Se-rich food. Adding a suitable amount of Se can promote the growth of plants, improve the quality of crops, enhance the stress resistance of crops, and improve the yield and Se content of crops^10^. In addition, appropriate application amount of Se can also improve the osmotic regulation ability of plants and reduce the toxic effects of heavy metals^11^.

*Moringa oleifera* is an edible medicinal plant, which is widely grown in Southeast Asia and Africa, and has attracted much attention as a "natural nutrition in tropical regions". All parts of *M. oleifera* have medicinal and nutritional functions. Its pods and leaves are rich in protein, amino acids, unsaturated fatty acids, bioactive substances and essential minerals^11–13^. The tender leaves and young pods can be used as vegetables, and leaf powder can also be added to flour to make *M. oleifera* noodles. Glucosinolates extracted from *M. oleifera* leaves have anticancer properties^14,15^, and flavonoids and polyphenols from the leaves have hypoglycemic, hypolipidemic and antioxidant effects^16^. *Oreochromis niloticus* (Nile tilapia fish) can effectively improve chlorpyrifos induced growth retardation, immune suppression, oxidative stress and DNA damage by eating *M. oleifera* leaves^17^. *M. oleifera* seeds are rich in unsaturated fatty acids and low molecular water-soluble proteins. The low molecular water-soluble proteins from *M. oleifera* seeds can be used as biological water purification agents^18,19^. *M. oleifera* flowers are a good source of ethyl carbamate and flavone and other substances, which have useful antimicrobial, anti-inflammation and anti-diabetes effects^20,21^. The agents-1,3-dibenzylurea and aurantiamide acetate extracted from *M. oleifera* root have anti-inflammatory and analgesic effects^22^, and the polysaccharide in the roots also has anti-inflammatory and antibacterial effects^23^. Therefore, *M. oleifera* roots can be used to treat low back pain, gout, rheumatism and lithiasis and other diseases^24^.

Plant roots are the main organ to absorb nutrients, and also the main place to synthesize amino acids, organic acids and various hormones in plants. Roots can also transport and distribute assimilates, so roots have a close relationship with the growth and development of other organs of plants. Root is the channel for nutrients and minerals to enter the plant, which will lead to elements such as Se and Cd to enter the food chain. In this study, we hypothesize that selenium could be enriched to reduce the Cd concentration in plants, and the current research wants to find a more effective method to reduce the Cd concentration of *M. oleifera* root. Thus, *M. oleifera* was cultivated with different concentrations of Na_2_SeO_3_ in soil to assess the effect of Se and Cd accumulation in the roots affected by exogenous Se.

## Results

### Cd and Se accumulation in *M. oleifera* roots

**Figure 1:**
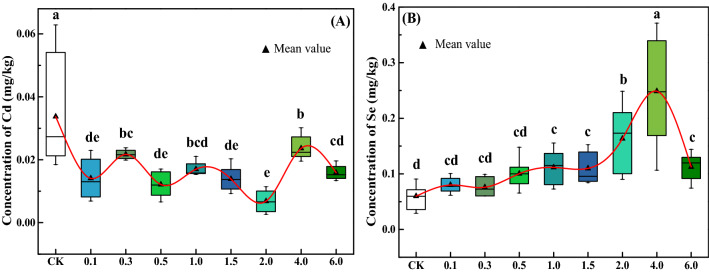
Box plots of Cd (**A**) and Se (**B**) concentrations in roots of *M. oleifera*. Boxes with different lowercase letters indicate significant differences in the mean between different treatments (*p* < 0.05).

ICP-MS results (Fig. 1) showed that the application of all concentration of Na_2_SeO_3_ significantly reduced the concentration of Cd (*p* < 0.05) whereas significantly enhanced concentration of Se in roots of *M. oleifera* (*p* < 0.05) than control sample. When Na_2_SeO_3_ was added to the soil less than 4.0 mg/kg, the concentration of Se accumulation in the root increased with the increase of the added amount. The concentration of Se decreases significantly (*p* < 0.05) while added above 4.0 mg/kg. Compared with the control group, Se increased 27–315% and Cd decreased 30–80% in the roots of *M. oleifera* with treatment of Na_2_SeO_3_ in soil.

### FTIR spectroscopy analysis

**Figure 2:**
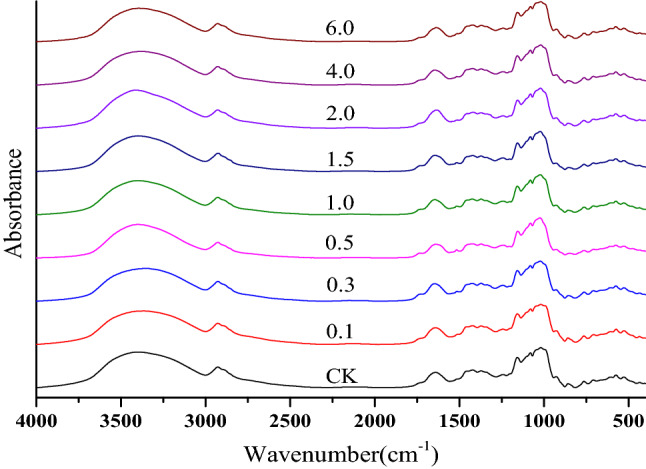
FTIR spectra of cultivated *M. oleifera* roots with Na_2_SeO_3_ concentration of 0, 0.1, 0.3, 0.5, 1.0, 1.5, 2.0, 4.0 and 6.0 mg/kg.

FTIR spectra of *M. oleifera* roots are shown in Fig. 2. O–H or N–H stretching vibration was at 3390 cm^−1^. The peaks around 2927 and 2884 cm^−1^ were assigned to methyl and methylene stretching vibrations^25^. The absorption peak of the vibration of the saturated ester groups compounds appeared around 1740 cm^−1^^26^ and the peak at 1643 cm^−1^ which was ascribed to the C = O stretching vibrations of carboxylic anions, hemicelluloses or amide groups in proteins^27^. The peak around 1517 cm^−1^ was attributed to lignin^28^. Carboxylate vibration was at 1421 cm^−1^^29^. The peaks around 1241 and 859 cm^−1^ were due to the S–O stretching vibrations^30^. The peaks at 1160, 1079 and 1020 cm^−1^ suggested the presence of hemicelluloses^28^. The peak around 929 cm^−1^ was ascribed to the vibration of β-glycoside^23^ and the peak at 765 cm^−1^ due to the vibration of α-glycoside^31^. The peak of S–O in the control sample was at 1238 cm^−1^. After the addition of exogenous Se, the peaks hifted to 1242 cm^−1^. Therefore, the addition of exogenous Se may affect S in roots of *M. oleifera*.

### PCA of spectra

**Figure 3:**
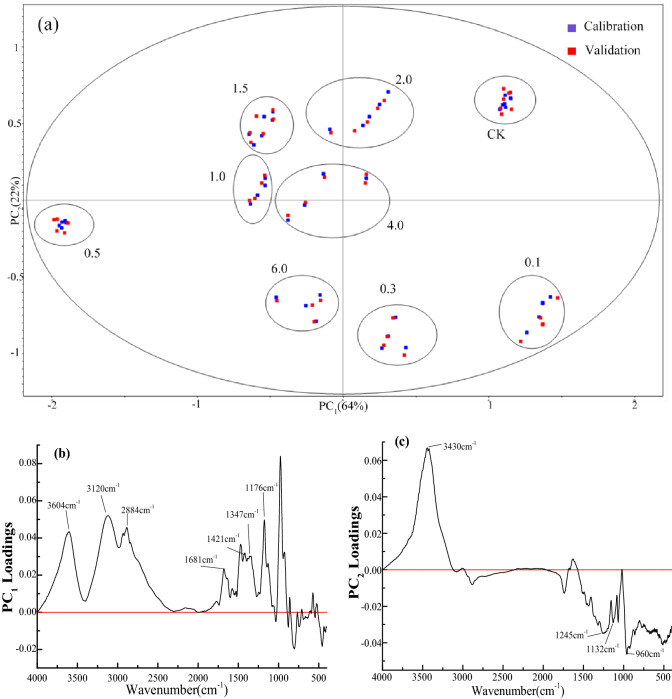
PCA scores plot of FTIR spectra of *M. oleifera* roots cultivated at different concentrations of Na_2_SeO_3_: (**a**) PCA scores plot; (**b**) PC_1_ loadings plot; (**c**) PC_2_ loadings plot.

PCA was used to reduce the dimensionality of the spectral data to understand the possible sources of the explained differences. From the PCA score plot (Fig. 3a), it can be seen that the FTIR spectra of *M. oleifera* root cultivated under different conditions could be accurately separated.

The loadings plot was examined to establish possible sources of variance within the spectra, and several areas of high variance were identified. Therefore, the loading plot of PCA can be used to explain the changes in the composition of *M. oleifera* roots cultivated under different conditions. According to PC_1_ loadings plot (Fig. 3b), there are strong positive weighted peaks around 3604, 3120 and 2884 cm^−1^, which are related to the stretching vibration of carboxyl and methyl groups. A positively weighted peak at 1681 cm^−1^, was due to hemicelluloses and amide^27^. The positively weighted peaks around 1421 and 1347 cm^−1^ were related to carboxylate radical^29^, and the positively weighted peak at 1176 cm^−1^ due to hemicelluloses^28^. Therefore, the loadings plot of PC_1_ is related to the changes of carboxylate and hemicellulose in the roots, and proteins may also be involved. The load of PC_2_ Fig. 3c has a strong positive weighted peak around 3430 cm^−1^, which is related to O–H or N–H stretching vibration. The negatively weighted peak at 1245 cm^−1^, was due to amide III, and the negative weighted peaks around 1132 and 960 cm^−1^ were related to the PO_4_^3−^ stretching vibration^31,32^. Therefore, the loadings plot of PC_2_ is related to the changes of PO_4_^3−^ and proteins in roots of *M. oleifera*. The results of PCA showed that the main sources of FTIR difference in *M. oleifera* roots were carboxylate, PO_4_^3−^, hemicellulose and proteins.

### PLSR analysis

**Figure 4:**
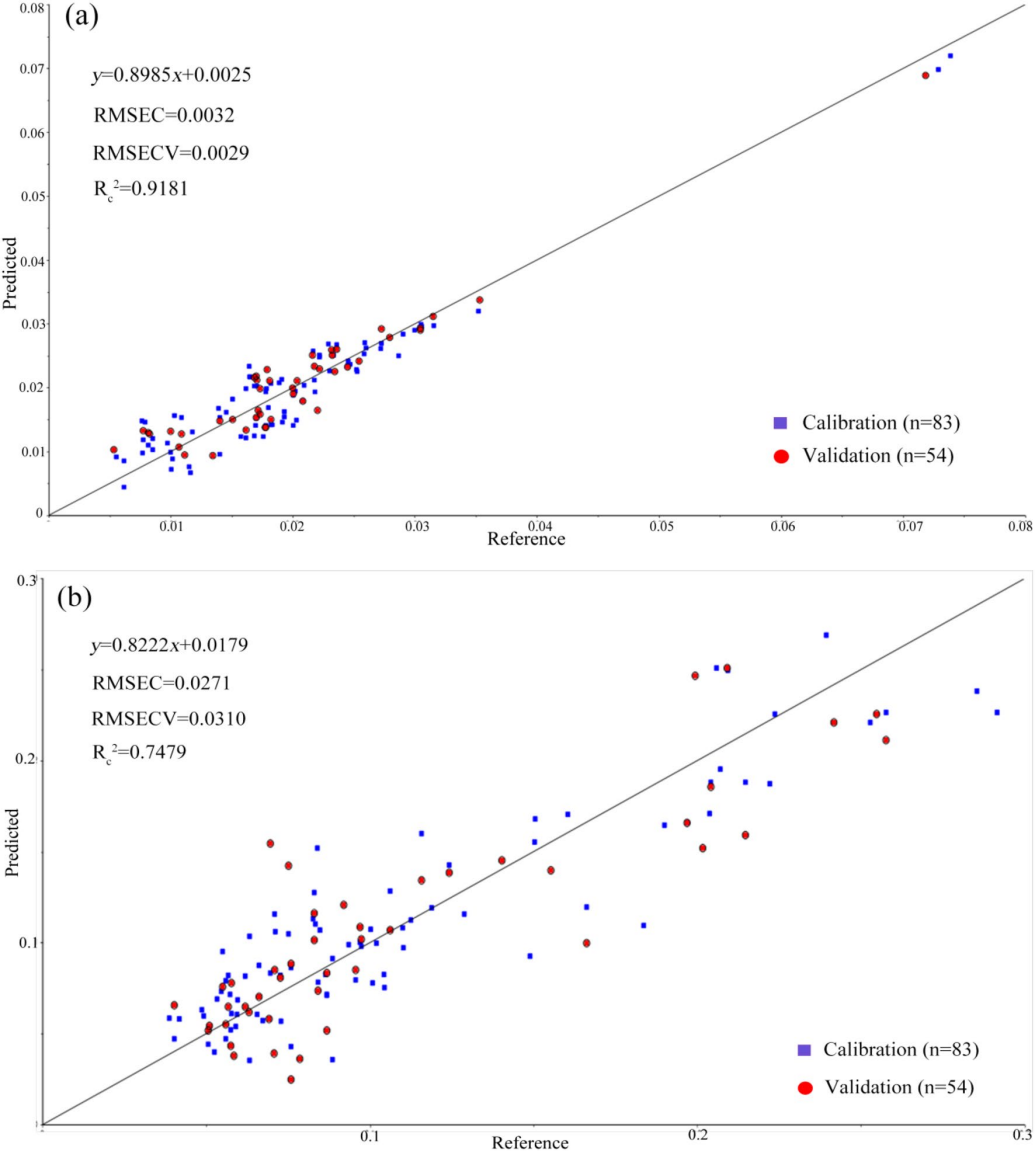
PLSR prediction model for Cd (**a**) and Se (**b**). “RMSEC”:root mean square error of calibration; “RMSECV”:root mean square error of cross validation.

**Figure 5:**
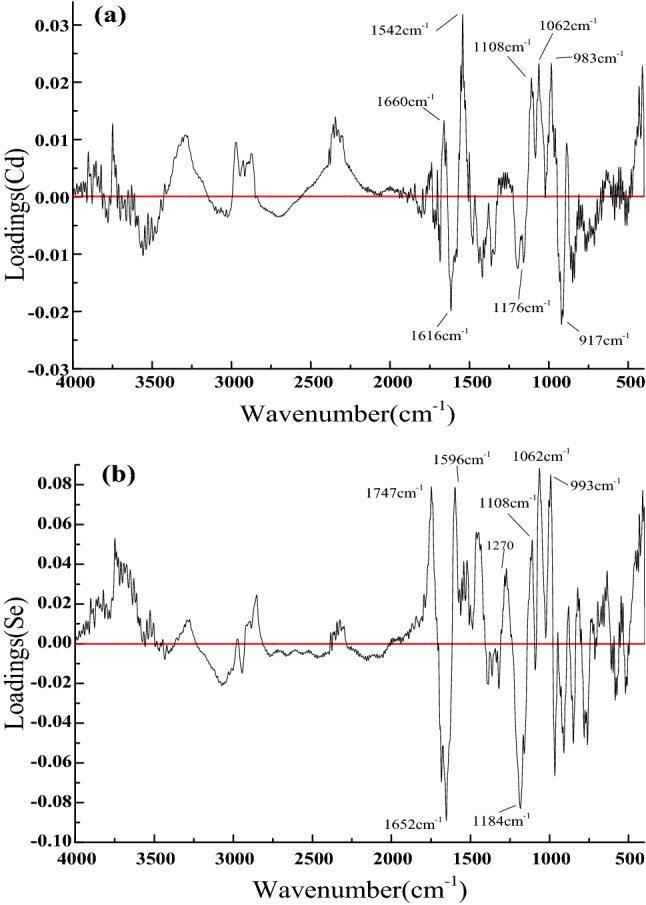
Loadings plot PLSR prediction model for Cd (**a**) and Se (**b**).

In order to understand the effect of related components in the root of *M. oleifera* on the accumulation of Cd and Se, the PLSR analysis of Cd and Se was established by infrared spectra (4000 ~ 400 cm^−1^), as shown in Fig. 4. The determination coefficients (R_c_^2^) for the PLSR analysis of Cd and Se were 0.9181 and 0.7479 respectively. Figure 5a shows the loadings plot of the wavenumber weight in the PLSR analysis of Cd. There are positive weighted peak around 1660 cm^−1^ and negative weighted peak around 1616 cm^−1^, the two peaks are related to amide I in the protein. The positive weighted peak around 1542 cm^−1^ is related to amide II. There are positive weighted peaks around 1176, 1108 and 1062 cm^−1^ which are related to hemicellulose. Therefore, the content of Cd in roots of *M. oleifera* might be affected by protein and hemicellulose. Figure 5b shows the loadings plot of the wavenumber weight in the PLSR analysis of Se. The positive weighted peak around 1747 cm^−1^ was related to hemicelluloses. The negative weighted peak at 1652 cm^−1^, indicative of carbonyl groups, hemicelluloses and amide, and the positive weighted peak around 1596 cm^−1^ is related to amide II of protein. The positive weighted peak around 1270 cm^−1^ is related to guaiacyl ringin in lignin^33^. The positive weighted peaks around 1108 and 1062 cm^−1^ and the negative weighted peaks around 1184 cm^−1^ are related to hemicellulose, while the positive weighted peaks around 993 cm^−1^ are related to polysaccharide vibration. Therefore, the content of Se in roots of *M. oleifera* might be affected by protein, hemicellulose, lignin and polysaccharide.

## Discussion

In this study, the cultivation of *M. oleifera* root by adding Na_2_SeO_3_ significantly increased the Se concentration. Previous studies have shown that Moringa has the exceptional ability to extract Se from the soil and accumulate it in the leaves, which is significantly higher than other plants^34,35^, and it probably also applies to roots. Plants absorb Se from the environment mainly via the roots. Selenite absorbed into the roots will be transported to various parts of the plant, but the selenite retained in the roots was higher than those transported to other parts, so the Se content in the roots was generally higher^36^.

Plant plasma membrane can maintain normal intracellular homeostasis and nutrition, and participate in the perception and response to various environmental stimuli, while the protein of plant plasma membrane plays an important role in response to the external environment. The results of FTIR and PCA showed that when different concentrations of Na_2_SeO_3_ were used to cultivate *M. oleifera*, the PO_4_^3−^ and protein in roots of *M. oleifera* were changed. This is due to the perception of plant roots to the external environment, mainly by reversible phosphorylation of the sensing protein, and using the membrane related G-protein, polyphosphoinositide signal pathway and other signal pathways for transmission^37^. Some studies have also found that the active absorption of Se is regulated by phosphate transporters^38^. Se can bind to sulfhydryl groups in certain proteins and inhibit Cd from entering cells^39^. Therefore, adding different concentrations of exogenous Se can change the growth environment of roots of *M. oleifera*. By regulating the abundance of proteins related tochannels, transporters and membrane vesicles transport, the root cells can promote or inhibit the absorption and transport of substances.

The absorption and accumulation of elements in plants are influenced by external environment (such as pH, humidity and temperature of soil), the regulation of transcription factors and the expression of related genes. bHLH transcription factors and jasmonic which are widely found in plants, play an important role in plant growth and secondary metabolite synthesis^40^. The bHLH transcription factors can increase the tolerance of Arabidopsis to Zn and Ni^41^, and also affect the response of plants to Cd absorption^42^. Jasmonic can improve the activity of stress resistant enzyme, and then promote the synthesis of alkaloids, flavonoids and antioxidants^40^. Previous studies have found that exogenous Se can up regulate the gene expression of hormone synthetase in plants, promote the synthesis of hormones such as jasmonate or methyl jasmonate, thereby inducing plants to absorb Se externally and increase the amount of Se^43^. Na_2_SeO_3_ enabled strawberry plants to improve the activity of antioxidant enzyme glutathione reductase and the activity of l-galactono-1, 4-lactone dehydrogenase responsible for the biosynthesis of ascorbate, to fight against cadmium stress^10^.

When Na_2_SeO_3_ was added to *M. oleifera*, the carboxyl group, hemicellulose and lignin in Moringa root changed, and the concentration of Cd in the root was significantly reduced (*p* < 0.05). According to the analysis of PLSR, the concentration of Cd was related to hemicellulose, which was consistent with the results of Guo et al.^28^. Root Cd mainly exists in the polysaccharides of the cell wall, which is attributed to the binding effect of the carboxyl and carboxylate groups in hemicellulose on Cd ions^44^. Therefore, exogenous Se caused the changes of hemicellulose and protein in the roots of *M. oleifera*, and effectively reduce the absorption of Cd. It has been reported that exogenous Na_2_SeO_3_ changes the number of cells per unit area of xylem in the root^45^, and increases the content of pectin and hemicellulose in the cell wall of the root^46^, the results of these studies were consistent with the results of our study. Exogenous Se changed the polysaccharides in roots, and affected the concentration of Cd in the roots.

## Materials and methods

### Cultivation of *M. oleifera* and experimental design

*M. oleifera* cultivation experiments were conducted from March 2017 to July 2018 in Xishuangbanna, Yunnan, China (101°25′N, 21°41′E). The soil for cultivation was taken from the acidic red soil locally in Xishuangbanna and collected from the 0 to 20 cm soil layer. The plant residue were removed from the soil and passed through a 10 mesh sieve after air-drying. Each pot used for cultivation was filled with 5 kg of soil. The basic properties of soil were shown in Table 1.

**Table 1:** Chemical characterization of red soil.

Properties	Amount	Properties	Amount (mg/kg)
Soil organic matter	4.34%	Mg	2602
pH	5.9	Na	371.7
Al	58,770 mg/kg	P	406.2
Cd	1.171 mg/kg	Se	1.90
Fe	29,500 mg/kg	Si	6.15
K	7616 mg/kg	Zn	184

The detected elements were total.

*M. oleifera* seed was obtained from Yunnan Manze Biotechnology Co., Ltd., Chian. Since selenite tends to accumulate more selenium in the roots of plants, this study applied selenite to cultivate *M. oleifera*. Eight experimental groups with Na_2_SeO_3_ and one blank control group were set up, the concentrations of Na_2_SeO_3_ in soil were 0, 0.1, 0.3, 0.5, 1.0, 1.5, 2.0, 4.0 and 6.0 mg/kg, respectively. Each treatment had three parallel experiments, 27 pots in total. Two seeds were planted in each pot and growing under natural light and temperature conditions. After 16 months, the plants were harvested and divided into leaves, stems and roots. The roots were washed with tap water and deionized water and dried to constant weight in a drying oven (50 °C), and then digested and analyzed.

### Measurement of total Se and Cd in dry matter

Three parallel experiments were carried out for each *M. oleifera*, and three blank groups of samples were set. 10 mL 69% HNO_3_ and 1 mL 70% HClO_4_ were used for digestion of samples (0.50 g). For digestion, a high-performance graphite furnace digestion system (DigiBlock ED54-iTouch, China) equipped with advanced composite PTFE vessels was used. The decomposition of organic matter was carried out at atmospheric pressure. When the rest digested solutions were clear with a volume of about 3 mL, 1 ml of 2% HNO_3_ and deionized water was used to adjust the samples to constant volume (25 mL). The total Cd and Se concentration was measured by ICP-MS (Elan DRC-e, Perkin Elmer, USA). In order to validate the methods, the standard reference materials soybean (GBW10013, China) were used as reference materials to assess the experimental procedures. The standard values of Cd and Se in reference materials were 0.011 and 0.022 mg/kg, respectively. The Cd and Se values of the reference materials measured by ICP-MS were 0.012 ± 0.003 and 0.024 ± 0.004 mg/kg (n = 3), respectively. Therefore, recoveries of Cd and Se in samples ranged from 92 to 109% and 95 ~ 112% respectively.

### Detection and analysis methods of FTIR spectroscopy

Infrared spectra were acquired using FTIR Spectroscopy (Frontier, Perkin Elmer, USA) equipped with a DTGS detector. All spectra were recorded in the range of 4000–400 cm^−1^ with a 4 cm^−1^ resolution and 16 scans. All samples were measured by KBr pellet method. The interferences of H_2_O and CO_2_ as well as KBr background were subtracted automatically when scanning. Quadruplicate spectra were collected for each sample. The average spectra were used for PCA and PLS regression analysis performed by using The Unscrambler X 10.4 software.

## Conclusion

In conclusion, exogenous Se significantly increased the content of Se in roots of *M. oleifera*, while significantly reduced the content of Cd. As our present study was only utilized the roots of *M. oleifera*, the effects of exogenous Se on the leaves of *M. oleifera* may be different. Therefore, it is necessary to further study the effect of exogenous Se on Cd and Se accumulation in *M. oleifera*. Even so, the results of this study still provide information for the roots of *M. oleifera* as a Se-enriched product.
